# Hypoparathyroidism-related health care utilization and expenditure during the first postoperative year after total thyroidectomy for cancer: a comprehensive national cohort study

**DOI:** 10.3389/fendo.2023.1193290

**Published:** 2023-06-28

**Authors:** Fares Benmiloud, Christine Le Bihan, Stanislas Rebaudet, Patricia Marino, Philippe-Jean Bousquet, Elsa Bouée-Benhamiche

**Affiliations:** ^1^ Department of Endocrine Surgery, Hôpital Privé de Provence, Aix-en-Provence, France; ^2^ Department of Endocrine Surgery, Hôpital Européen Marseille, Marseille, France; ^3^ Department of Health Data and Assessment, French National Cancer Institute (Institut National du Cancer, INCa), Boulogne-Billancourt, France; ^4^ Aix-Marseille Institute of Public Health, Institut des sciences de la santé publique d’Aix-Marseille (ISSPAM), Sciences économiques et sociales de la santé & traitement de l’information médicale Unité mixte de recherche (SESSTIM), Aix-Marseille Univ, Institut national de la santé et de la recherche médicale (INSERM), Institut de recherche pour le développement (IRD), Marseille, France; ^5^ Institut Paoli-Calmettes Sciences économiques et sociales de la santé & traitement de l’information médicale Unité mixte de recherche (SESSTIM UMR) 1252, Institut national de la santé et de la recherche (INSERM), Institut de recherche pour le développement (IRD), Aix Marseille University, Marseille, France

**Keywords:** postoperative hypocalcemia, economic evaluation, thyroidectomies, health care expenditure, thyroid cancer

## Abstract

**Objectives:**

Hypoparathyroidism is the most common complication of total thyroidectomy for cancer, and requires calcium and/or vitamin D supplementation for an unpredictable period of time. The additional cost associated with this complication has not hitherto been assessed. The aim of this study was to assess the economic burden of postoperative hypoparathyroidism after total thyroidectomy for cancer in France.

**Methods:**

Based on the French national cancer cohort, which extracts data from the French National Health Data System (SNDS), all adult patients who underwent a total thyroidectomy for cancer in France between 2011 and 2015 were identified, and their healthcare resource use during the first postoperative year was compared according to whether they were treated postoperatively with calcium and/or vitamin D or not. Univariate and multivariate cost analyses were performed with the non-parametric Wilcoxon test and generalized linear model (gamma distribution and log link), respectively.

**Results:**

Among the 31,175 patients analyzed (75% female, median age: 52y), 13,247 (42%) started calcium and/or vitamin D supplementation within the first postoperative month, and 2,855 patients (9.1%) were still treated at 1 year. Over the first postoperative year, mean overall and specific health expenditures were significantly higher for treated patients than for untreated patients: €7,233 vs €6,934 per patient (p<0.0001) and €478.6 vs €332.7 per patient (p<0.0001), respectively. After adjusting for age, gender, Charlson Comorbidity index, ecological deprivation index, types of thyroid resection, lymph node dissection and complications, year and region, the incremental cost of overall health care utilization was €142 (p<0.004).

**Conclusion:**

Our study found a significant additional cost in respect of health expenditures for patients who had hypoparathyroidism after thyroidectomy for cancer, over the first postoperative year. Five-year follow-up is planned to assess the impact of more severe long-term complications on costs.

## Introduction

Postoperative hypoparathyroidism, the most frequent complication after total thyroidectomy for cancer, is caused by post-surgical parathyroid gland failure. The resultant hypocalcemia generally requires calcium +/- vitamin D supplementation. This supplementation can be discontinued after recovery, in the case of ‘temporary’ postoperative hypoparathyroidism, but after six months to one year, the condition may be considered as ‘permanent’. The rates reported in most recent large-scale and national cohort studies in different countries range from 20 to 40% for temporary hypoparathyroidism, and from 5 to 12% for permanent hypoparathyroidism (1–5).

In the course of acute and chronic supplemented postoperative hypoparathyroidism, complications may occur and lead to repeated medical visits, such as intravenous calcium extravasations (6), rehospitalizations for calcium disorders, as well as, in the long term, neurological, psychiatric, bone-related (7), cardiac and renal morbidity (8), and excess mortality (9).

The clinical burden of hypoparathyroidism has been studied, but without any estimate of the associated costs. The economic evaluations in postoperative hypoparathyroidism have been designed to compare calcium +/- vitamin D supplementation strategies (10–15). Thus, to date, no large-scale study has assessed the economic impact of this complication. This impact can be directly due to specific patient care associated with the provision and monitoring of drug supplementation and with the management of its potential consequences, such as longer hospital stays, regular and repeated biological tests and/or medical consultations, but also with indirect care expenditure that cannot reasonably be predicted, such as increased daily allowances on account of sick leave, etc.

Since 2010, the French “Cancer Cohort” has compiled the exhaustive individual sociodemographic and medical characteristics, hospital stays, and outpatient healthcare consumption for all patients who have had cancer in France, using data from the French SNIIRAM/SNDS repository, which is the national health insurance information system (16, 17). The amounts reimbursed by the national health insurance for all these healthcare consumptions are also recorded in this information system. This cohort, whose data have already been used to trace the care pathway of patients treated for breast cancer (18) or assess access to palliative care in France (19, 20), offers unique resources for an exhaustive evaluation, at a national level, of the health care expenditure of patients with thyroid cancer.

The aim of our study was to assess the economic burden of hypoparathyroidism for the first year post-total thyroidectomy for cancer in France, and to describe overall and specific health expenditures, as well as cost determinants.

## Methods

### Data sources

This observational study was based on the French national cancer cohort, which includes all patients diagnosed or treated for cancer since 2010. This cohort, described in detail elsewhere (17), is extracted from the large-scale French National Health Data System (SNDS) (21).

Briefly, SNDS is a national database collecting healthcare consumption and reimbursement claims covering 99% for the French population (i.e., 67 million people). It includes demographic data (e.g., sex, date of birth, date of death if applicable, health insurance scheme), hospitalization data (diagnoses, medical procedures, expensive drugs and medical devices), outpatient care data (drugs dispensed, lab tests, procedures and services). Sick leave allowances and disability allowances are also entered in SNDS.

The Diagnosis codes were recorded based on the International Classification of Diseases – 10^th^ revision, ICD-10 (World Health Organization) (22). Procedures were recorded with the CCAM classification (*classification commune des Actes Médicaux*) (23). Medicinal products were identified with the Anatomical Therapeutic Classification (ATC) code (24). Laboratory assays were identified with the national laboratory test coding table (NABM) (25).

### Ethics

This study was authorized by the French Data Protection Agency (Commission nationale de l’informatique et des libertés—Cnil (26)) n°2019-082 and 2019-083.

### Study population/cohort

In this study, we included all adult patients who underwent, between 2011 and 2015, total thyroidectomy or completion thyroidectomy for cancer (ICD-10 codes: C73 or D093 or D440 or E070 or D448), with or without central and/or lateral lymph node dissection (CCAM codes: KCFA005 or KCFA007 or KCFA002 or KCFA003 or KCFA006 or KCMA001). We excluded patients who were taking calcium and/or active vitamin D up to the last 30 days before the procedure (except if there was only one drug delivery in the 90 days preceding the procedure), as well as patients with a previous history of parathyroid pathology or resection (see codes in appendix).

In this study we used the ATC codes A12AA (Calcium), A12AA01 (Calcium phosphate), A12AA02 (Calcium glubionate), A12AA03 (Calicm gluconate), A12AA04 (Calcium carbonate), A12AA20 (different Calcium salts in association), A12AX (combinations of Calcium with vitamin D and/or other drugs) to search for what we defined as « Calcium treatments ». Our definition of « Vitamin D treatment » was selective: we searched either for Calcitriol (ATC code A11CC03) or for Alfacalcidiol (ATC code A11CC04)

We divided patients into two groups according to whether they could be considered as having had postoperative hypoparathyroidism or not. Group 1 included patients who started calcium and/or vitamin D supplementation within the first postoperative month and/or were hospitalized for severe hypocalcemia at any time in the first year (ICD-10 code E83.51: Code for hypocalcemia). Group 2 included the other patients.

Subgroups were also defined to better characterize potential hypoparathyroidism. Subgroup 1A consisted of patients treated during the first postoperative month and who continued the treatment continuously during the first postoperative year (patients treated for probable permanent hypoparathyroidism), subgroup 1B consisted of patients treated during the first postoperative month and who discontinued their treatment during the first postoperative year (patients treated for probable temporary hypoparathyroidism), and subgroup 1Z consisted of patients who were hospitalized with hypocalcemia <1.5mmol/l (ICD-10 code E83.51) without any treatment during the first postoperative month (patients with hypoparathyroidism probably untreated or undetected during the first postoperative month). Subgroup 2C consisted of patients never treated during the first postoperative year (patients without hypoparathyroidism), subgroup 2D consisted of patients untreated during the first postoperative month, but who started treatment in the second or third postoperative month (patients with indeterminate postoperative parathyroid status), and subgroup 2E consisted of patients untreated during the first postoperative month, who started treatment after the third postoperative month (patients treated for a reason probably unrelated to hypoparathyroidism).

### Variables of interest and outcomes

The study population was described using the following criteria: age, gender, chronic comorbidities at any time in the last 365 days prior to thyroidectomy (list of 17 included in the Charlson comorbidity index) (27–30), French deprivation index (31), year of surgery, length of thyroidectomy hospital stay, geographical area of thyroidectomy hospital stay, type of thyroid resection (total thyroidectomy, completion thyroidectomy, complex total thyroidectomy), type of lymph node dissection performed (none, central compartment, lateral compartment), radioactive iodine (RAI) treatment, other complications (laryngeal, hemorrhagic, infectious and cutaneous complications), and rehospitalization for calcium disorders (at any time during the first postoperative year for the last three criteria).

Costs were estimated from the payer’s perspective (French National Health Insurance).

All healthcare consumption reimbursed by the payer during the first postoperative year were identified in the database, i.e., from the end of surgery hospitalization up to day 365 after the date of surgery, or until the date of death, whichever occurred first.

Inpatient (hospitalization) care costs based on the Diagnosis-Related Group tariffs and drugs or medical devices billable in addition to the DRGs in public and private hospitals, outpatient care costs (drugs, biological tests, imaging procedures, total medical consultations, transportation and other procedures, such as physiotherapy or speech therapy, etc.), and indirect costs (all-cause sick leave daily allowance and invalidity allowance) were included in the overall reimbursed healthcare expenditures.

Some health expenditure items thought to be specifically related to hypoparathyroidism were also described, and included some specific medications, medical consultations, bioassays, imaging and rehospitalization for calcium disorders (other than severe hypocalcemia). Specific drug prescriptions included conventional treatments prescribed to limit the long-term harm of hypoparathyroidism: calcium, active vitamin D, magnesium, thiazide diuretics, and phosphate binders. The specific medical consultations concerned doctors likely to have been directly involved in the treatment (general practitioner, endocrinologist, general/visceral surgeon, ENT surgeon) or its complications (plastic surgeon, rheumatologist, nephrologist, urologist, psychiatrist, cardiologist). Specific bioassays concerned tests associated with the monitoring of hypoparathyroidism and its potential renal complications: calcium, phosphorus, parathormone, 25-(OH)-vitamin D (D2+D3), plasma magnesium, urea, creatinine, urine calcium level, urine creatinine level, creatine clearance (32). Specific imaging tests were those associated with monitoring potential complications of treated hypoparathyroidism: urinary tract ultrasound, brain CT and/or MRI, skull X-ray and bone densitometry (32). Specific rehospitalizations for calcium disorders also provide information on monitoring of potential parathyroid-related complications. ATC, NABM, CCAM and ICD-10 codes used to collect drugs, bioassays, imaging and hospitalization are shown in the Appendix.

### Statistical analysis

Categorical variables were described with frequency and percentage, continuous variables with mean and standard deviation ( ± SD) or median.

Univariate cost analyses were performed with the non-parametric Wilcoxon test, multivariate analyses were carried out using generalized linear models (GLMs) with log link and gamma distribution. Incremental expenditures associated with hypocalcemia and covariates were estimated from respective regression coefficients (independent differentials) in GLM including all the following covariates: age (1^st^ quartile to 4^th^ quartile), gender, Charlson comorbidity index (0, 1 or 2, > 2), French deprivation index (from 1^st^ quintile: the least deprived quintile to the 5^th^ quintile: the most deprived quintile), year of surgery (2011 to 2015), geographical area of thyroidectomy hospital stay (13 administrative areas), type of thyroid resection (total thyroidectomy, completion thyroidectomy, complex total thyroidectomy), type of lymph node dissection (none, central compartment, lateral compartment), laryngeal complications, hemorrhagic complications, infectious complications, cutaneous complications (complications at any time during the first postoperative year).

Statistical tests were two-sided, a p-value < 0.05 was considered as significant.

All statistical analyses were performed using World Programming System 4.02.

## Results

### Selection of the study population

**Figure 1:**
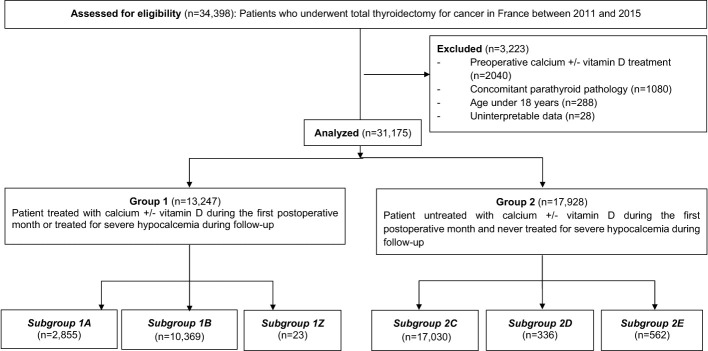
Flow chart.

Between January 1st, 2011, and December 31st, 2015, 34,398 patients underwent total thyroidectomy for thyroid cancer in France. After taking into account for exclusion factors (preoperative calcium or vitamin D-calcium therapy for 2,040 patients, associated parathyroid pathology for 1,080 patients, age <18 years for 288 patients, and uninterpretable data for 28 patients), 31,175 patients were analyzed, as detailed in the flow chart, **Figure 1**.

### Patient characteristics

In total, out of the 31,175 patients analyzed, 13,247 patients (42.49%) were deemed to have postoperative hypoparathyroidism on account of calcium or vitamin D+calcium replacement therapy started within the first postoperative month (n=13,224: 4,344 with calcium alone, and 8,880 with vitamin D+calcium) or hospitalization for severe hypocalcemia at any time in the first year (n=23). They were included in Group 1.

The remaining 17,928 patients (57.51%) were assigned to Group 2. The main characteristics of both groups were described (**Table 1**).

**Table 1:** Patient characteristics.

	Group 1	Group 2
	N=13,247	N=17,928
Mean (SD) age in years:	49.8 (14.8)	52.7 (14.9)
Sex, n (%):		
Female	10,541 (79.6)	12,738 (71.1)
Male	2,706 (20.4)	5,190 (28.9)
Comorbidities^(1)^, n (%):		
Vascular-cerebral disease	73 (0.6)	136 (0.8)
Connective tissue disorder	93 (0.7)	121 (0.7)
Dementia	12 (0.1)	15 (0.1)
Complicated diabetes	59 (0.5)	126 (0.7)
Uncomplicated diabetes	665 (5.0)	1,249 (7.0)
Hemiplegia	19 (0.1)	22 (0.1)
Heart failure	32 (0.2)	88 (0.5)
Mild liver disease	40 (0.3)	61 (0.3)
Moderate to severe liver disease	10 (0.1)	7 (0.0)
Myocardial infarction	146 (1.1)	277 (1.6)
Metastatic solid tumor	34 (0.3)	48 (0.3)
Moderate to severe kidney disease	42 (0.3)	65 (0.4)
Respiratory disease	74 (0.6)	105 (0.6)
Non-cutaneous tumor	977 (7.4)	1,469 (8.2)
Vascular ulcer	1 (0.0)	1 (0.0)
Peripheral vasculopathy	72 (0.5)	128 (0.7)
HIV-AIDS	12 (0.1)	28 (0.2)
Ecological deprivation index, n(%):		
1^st^ quintile (least deprived)	2,769 (20.9)	3,603 (20.1)
2^nd^ quintile	2,780 (21.0)	3,449 (19.2)
3^rd^ quintile	2,614 (19.7)	3,363 (18.8)
4^th^ quintile	2,509 (18.9)	3,382 (18.9)
5^th^ quintile (most deprived)	2,219 (16.8)	3,512 (19.6)
Type of thyroid resection, n (%):		
Total thyroidectomy	12,072 (91.1)	15,107 (84.3)
Completion thyroidectomy	1,029 (7.8)	2,604 (14.5)
Complex total thyroidectomy	146 (1.1)	217 (1.2)
Type of lymph node dissection, n (%):		
None	7,561 (57.1)	12,226 (68.2)
Central compartment	2,891 (21.8)	2,887 (16.1)
Lateral compartment	2,795 (21.1)	2,815 (15.7)
Length of stay in days, mean (SD):	3.5 (2.4)	3.4 (3.4)
Complications^(2)^, n (%):		
Laryngeal	3,079 (23.2)	3,673 (20.5)
Hemorrhagic	260 (2.0)	380 (2.1)
Infectious	144 (1.1)	230 (1.3)
Cutaneous	93 (0.7)	127 (0.7)
Radioactive iodine therapy^(2)^, n (%):	7,951 (60.0)	10,085 (56.3)
Rehospitalizations for calcium disorders^(2)^, n (%):	281 (2.1)	56 (0.3)

Group 1 consisted of patients with hypoparathyroidism;

Group 2 consisted of patients without hypoparathyroidism or with indeterminate parathyroid status;

Length of stay corresponded to the hospitalization for total or completion thyroidectomy

(1) anytime in the last 365 days prior to thyroidectomy

(2) anytime between Day 0 and Day 365.

Among the 13,224 patients who started calcium and/or vitamin D supplementation within the first postoperative month, 2,855 (22%) were still treated at one year (Subgroup 1A). Of the other 10,369 patients who discontinued their treatment before 1 year (Subgroup 1B), 9,530 (92%) were treated for less than 6 months.

Among the 17,928 patients without any supplementation within the first postoperative month (Group 2), 17,030 patients (55% of the patients of this study) took no treatment during the first year (Subgroup 2C), 336 patients started supplementation in the second or third postoperative month (subgroup 2D, indeterminate postoperative parathyroid status), with 106 (32%) of these still treated at 1 year, and 562 patients started supplementation after the third postoperative month (subgroup 2E, indeterminate postoperative parathyroid status but probably not related to thyroid surgery), with 269 (49%) of these still treated at 1 year. Ultimately, 375 of these 898 patients with undetermined parathyroid status were still treated at 1 year.

**Figure 2:**
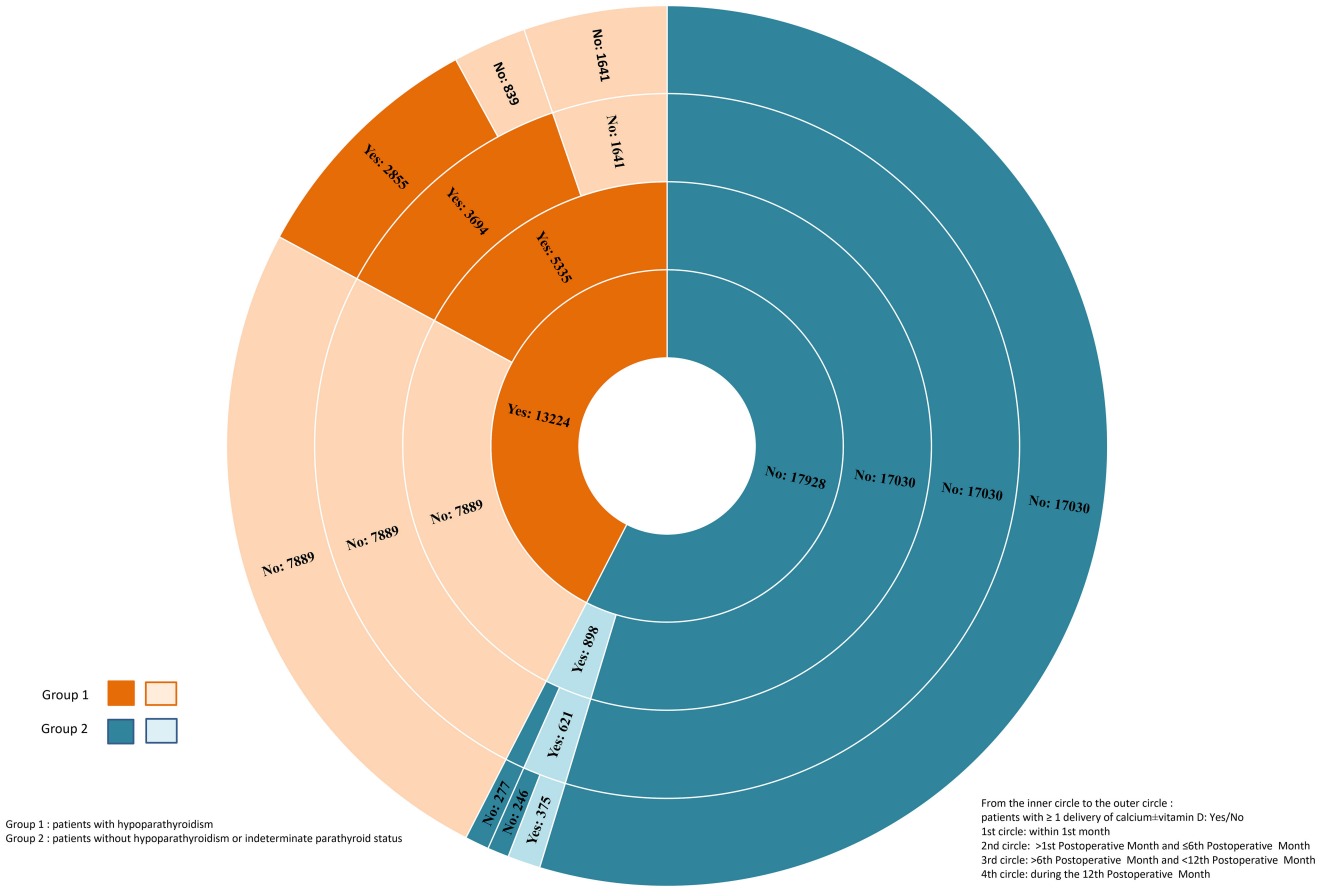
Duration of Supplementation by Time Intervals.

A representation of the duration of supplementation (≥1 calcium ± vitamin D delivery) by time intervals: within the first month, >M1 and ≤M6, >M6 and <M12, or at M12 accounts for late recoveries (**Figure 2**). For instance, patients still treated 1 year after thyroidectomy, from 9.1% (2,855) to 10.4% (3,230) of patients, can be found in the peripheral circle of the figure.

Regarding health care resource utilization within the first postoperative year, almost all patients received outpatient care, 70% of patients were hospitalized at least once: 9,523 patients (72%) in Group 1 versus 12,336 patients (69%) in Group 2; 91% had at least one specific bioassay: 12,886 patients (97%) in Group 1 versus 15 440 (86%) in Group 2; 46% received at least one specific drug: 1,069 patients (6%) in Group 2 versus all patients in Group 1, except 15 of 23 included because of hospitalization for severe hypocalcemia; imaging was observed in 8% of patients in each group; at least one rehospitalization for calcium disorders was observed in 277 patients (2.1%) in Group 1 versus 56 patients (0.3%) in Group 2. In addition, 37% of patients received daily allowances: 5,263 (40%) in Group 1 versus 6,259 (35%) in Group 2.

### Health expenditures

The overall health expenditure of the 31,175 patients during the first postoperative year was €95,809,106 in Group 1 and €124,315,208 in Group 2 (**Table 2**). The mean overall health expenditure per patient during the first year was €7,233 (±€9,256) in Group 1 and €6,934 (±€8,751) in Group 2, with an average difference of €298 per patient (p<0.0001). The average difference between the two groups in inpatient care costs was €23 (p<0.0001), the difference in outpatient expenses was €111 (p<0.0001), and the difference in indirect expenses was €164 (p<0.0001).

**Table 2:** Overall expenditures.

	Group 1	Group 2	1 vs 2	
	N=13,247	N=17,928	Δ	p-value
Outpatient care costs				
Total in euros	38,193,149	49,698,631		
Mean (SD) per patient in euros	2,883.2 (5,657.1)	2,772.1 (4,267.8)	111.0	<.0001
Median in euros (q1 – q3)	2,008 (1,139 – 3,175)	1,892 (1,017 – 3,077)		
Inpatient (hospitalization) care costs				
Total in euros	40,239,399	54,047,656		
Mean (SD) per patient in euros	3,037.6 (4,849.1)	3,014.7 (5,365.4)	22.9	<.0001
Median in euros (q1 – q3)	2,574 (0 – 3,382)	2,275 (0 – 3,175)		
Indirect costs				
Total in euros	17,376,559	20,568,921		
Mean (SD) per patient in euros	1,311.7 (3,002.1)	1,147.3 (2,929.8)	164.4	<.0001
Median in euros (q1 – q3)	0 (0 – 1,086)	0 (0 – 821)		
Total overall expenditures				
Total in euros	95,809,106	124,315,208		
Mean (SD) per patient in euros	7,232.5 (9,256.0)	6,934.1 (8,750.5)	298.4	<.0001
Median in euros (q1 – q3)	5,254 (2,706 – 8,284)	5,018 (2,177 – 7,852)		

**Group 1** consisted of patients with hypoparathyroidism;

**Group 2** consisted of patients without hypoparathyroidism or with indeterminate parathyroid status;

**1 vs 2:** comparison between Group1 and Group 2 on mean costs per patient;

**Δ**: Average difference ; univariate analysis : comparison using non-parametric Wilcoxon test;

**Indirect costs** included all-cause sick leave allowance and invalidity allowance.

Specifically, hypoparathyroidism-related health care expenditures during the first year were €6,339,607, with an average of €478.6 (€ ± 708.8) per patient in Group 1 versus a total of €5,964,120 and an average of €332.7 (€ ± 461.0) per patient in Group 2 (**Table 3**). This resulted in a mean difference between the groups of €145.9 per patient (p<0.0001), mainly due to drugs and rehospitalizations for calcium disorders.

**Table 3:** Specific expenditures.

	Group 1	Group 2	1 vs 2	
	N=13,247	N=17,928	Δ	p-value
Drugs				
Total in euros	969,836	43,199		
Mean (SD) per patient in euros	73.2 (127.2)	2.4 (26.5)	70.8	<.0001
Median in euro (q1 – q3)	21 (9 – 68)	0 (0 – 0)		
Bioassays				
Total in euros	345,682	207,4184		
Mean (SD) per patient in euros	26.1 (33.6)	11.6 (18.3)	14.5	<.0001
Median in euros (q1 – q3)	16 (6 – 33)	5 (2 – 14)		
Medical consultations				
Total in euros	4,201,541	5,388,646		
Mean (SD) per patient in euros	317.2 (284.7)	300.6 (291.6)	16.6	<.0001
Median in euros (q1 – q3)	247 (154 – 388)	238 (146 – 373)		
Imaging				
Total in euros	47,563	61,403		
Mean (SD) per patient in euros	3.6 (14.2)	3.4 (14.0)	0.2	0.1772
Median in euros (q1 – q3)	0 (0 – 0)	0 (0 – 0)		
Rehospitalization for calcium disorder				
Total in euros	774,985	263,455		
Mean (SD) per patient in euros	58.5 (591.7)	14.7 (347.1)	43.8	<.0001
Median in euros (q1 – q3)	0 (0 – 0)	0 (0 – 0)		
Total Specific Expenditures				
Total in euros	6,339,607	5,964,120		
Mean (SD) per patient in euros	478.6 (708.8)	332.7 (461.0)	145.9	<.0001
Median in euros (q1 – q3)	334 (206 – 541)	254 (156 – 398)		

**Group 1** consisted of patients with hypoparathyroidism;

**Group 2** consisted of patients without hypoparathyroidism or with indeterminate parathyroid status;

**1 vs 2**: comparison between Group1 and Group 2 on mean costs per patient;

**Δ**: Average difference ; univariate analysis : comparison using non-parametric Wilcoxon test;

**Specific expenditures** (after hospital discharge) were estimated for outpatients, except for rehospitalization related to calcium disorder;

**Drugs** included calcium, active vitamin D, magnesium, thiazides, phosphate binders;

**Bioassays** included calcium, phosphorus, parathormone, vitamin D (D2+D3), magnesium, urea, creatinine, urine calcium level, urine creatinine level, creatine clearance;

**Medical consultations** included general practitioner, endocrinologist, general/visceral surgeon, ENT, plastic surgeon, rheumatologist, nephrologist, urologist and psychiatrist;

**Imaging** included urinary tract ultrasound, brain CT and/or MRI, skull X-ray and bone densitometry;

**Rehospitalization** for calcium disorder included the following ICD-10 codes: E835, E8350, E8358.

To take into account potential confounders, a multivariate analysis was performed. After adjustment, the 1-year incremental cost in patients treated for postoperative hypoparathyroidism was estimated at €142 (p<0.004) (**Table 4**).

**Table 4:** Subgroup analysis on specific expenditures.

Subgroups:	1A	1B	1Z	2C	2D	2E	1A vs 1B		1A vs 2C	
	N=2,855	N=10,369	N=23	N=17,030	N=336	N=562	Δ	p	Δ	p
Outpatient care:										
Total in euros	10,733,131	27,319,071	140,947	45,927,918	1,394,458	2,376,256				
Mean per patient in euros (SD)	3,759.4 (6,138.0)	2,634.7 (5,488.7)	6,128.1 (6 635.8)	2,696.9 (4,172.4)	4,150.2 (5,701.3)	4,228.2 (5,545.8)	1,124.7	<.0001	1,062.5	<.0001
Median in euros Q1 Q3	2,535 1,563 3,943	1,865 1,039 2,977	3,323 1,316 10,645	1,868 1,000 3,036	2,460 1,362 3,967	2,449 1,453 4,550				
Inpatient (Hospitalization) care										
Total in euros	10,201,230	29,785,219	252,950	49,491,026	1,512,040	3,044,590				
Mean per patient in euros (SD)	3,573.1 (6,180.1)	2,872.5 (4,363.9)	10,997.8 (10,566.2)	2,906.1 (4,946.6)	4,500.1 (7,163.3)	5,417.4 (11,792.6)	700.6	<.0001	667	<.0001
Median in euros Q1 Q3	2,909 425 4,232	2,410 0 3,175	6,168 3,115 16,650	2,239 0 3,169	2,923 0 4,238	2,927 0 4,730				
Indirect costs										
Total in euros	4,422,574	12,934,640	19,345	19,571,835	295,920	701,166				
Mean per patient in euros (SD)	1,549.1 (3,416.1)	1,247.4 (2,875.5)	841.1 (2,545.4)	1,149.3 (2,933.7)	880.7 (2,446.8)	1,247.6 (3,069.3)	301.6	0.9668	399.8	<.0001
Median in euros Q1 Q3	0 0 1,169	0 0 1,074	0 0 0	0 0 831	0 0 511	0 0 589				
Overall :										
Total in euros	25,356,935	70,038,930	413,242	114,990,779	3,202,417	6,122,012				
Mean per patient in euros (SD)	8,881.6 (10,819.5)	6,754.7 (8,690.5)	17,967.0 (14,961.8)	6,752.3 (8,304.4)	9,531.0 (11 840.1)	10,893.3 (15,781.5)	2,126.9	<.0001	2,129.3	<.0001
Median in euros Q1 Q3	6,149 3,754 10,168	5,035 2,445 7,817	14,524 5,822 27,357	4,966 2,129 7,736	5,947 3,046 10,154	6,094 3,260 11,401				

**Subgroup 1A** consisted of patients treated during the first postoperative month and who continued treatment continuously during the first postoperative year;

**Subgroup 1B** consisted of patients treated during the first postoperative month and who discontinued treatment during the first postoperative year;

**Subgroup 1Z** consisted of patients who were hospitalized with hypocalcemia <1.5mmol/l (ICD-10 code E8351) without any treatment during the first postoperative month;

**Subgroup 2C** consisted of patients untreated during the first postoperative year;

**Subgroup 2D** consisted of patients untreated during the first postoperative month, but who started treatment in the second or third postoperative month;

**Subgroup 2E** consisted of patients untreated during the first postoperative month, who started treatment after the third postoperative month

**1A vs 1B** : comparison between Group1A and Group 1B on mean costs per patient;

**1A vs 2C** : comparison between Group1A and Group 2C on mean costs per patient;

Δ : Average difference ; univariate analysis : comparison using non-parametric Wilcoxon test ;

Indirect costs included all-cause sick leave allowance and invalidity allowance.

### Subgroup analysis

The mean differences between patients in Subgroup 1A (permanent hypoparathyroidism) and Subgroup 1B (temporary hypoparathyroidism) were €2,126.9 per patient (p<0.0001) for overall expenditures (**Table 5**), and €434.6 (p<0.01) per patient for specific expenditures (**Table 6**).

**Table 5:** Subgroup analysis on specific expenditures.

Subgroups:	1A	1B	1Z	2C	2D	2E	1A vs 1B		1A vs 2C	
	N=2,855	N=10,369	N=23	N=17,030	N=336	N=562	Δ	p value	Δ	p value
Drugs										
Total in euros	683,695	285,425	716	8,876	21,699	12,624				
Mean per patient in euros (SD)	239.5 (182.3)	27.5 (42.7)	31.2 (62.5)	0.5 (20.4)	64.6 (95.9)	22.5 (39.4)	212.0	<.0001	239.0	<.0001
Median in euros Q1 Q3	205 110 334	15 7 30	0 0 44	0 0 0	26 8 72	11 5 23				
Bioassays										
Total in euros	145,315	199,876	49,058	182,940.	10,040	14,439				
Mean per patient in euros (SD)	50.9 (48.9)	19.3 (23.8)	21.3 (30.0)	10.7 (16.8)	29.9 (36.3)	25.7 (30.6)	31.6	<.0001	40.2	<.0001
Median in euros Q1 Q3	37 20 66	12 5 25	12 0 28	5 1 14	19 8 39	17 7 35				
Medical consultations										
Total in euros	1,061,531	3,133,768	6,242	5,066,870	118,625	203,150				
Mean per patient in euros (SD)	371.8 (336.8)	302.2 (266.6)	271.4 (291.0)	297.5 (291.9)	353.1 (306.3)	361.5 (263.7)	69.6	<.0001	74.3	<.0001
Median in euros Q1 Q3	294 192 445	236 145 370	205 45 362	235 144 369	277 176 423	283 185 460				
Imaging										
Total in euros	12,670	34,820	73	55,230	1,554	4,619				
Mean per patient in euros (SD)	4.4 (16.3)	3.4 (13.5)	3.17 (10.6)	3.2 (13.6)	4.6 (15.4)	8.2 (22.0)	1.1	<.0001	1.2	<.0001
Median in euros Q1 Q3	0 0 0	0 0 0	0 0 0	0 0 0	0 0 0	0 0 0				
Rehospitalization for calcium disorder										
Total in euros	428,386	308,262	38,338	192,569	30,438	40,448				
Mean per patient in euros (SD)	150.1 (863.9)	29.7 (445.5)	1,666.9 (4035.3)	11.3 (314.8)	90.6 (604.0)	72.0 (784.5)	120.3	<.0001	138.7	<.0001
Median in euros Q1 Q3	0 0 0	0 0 0	0 0 0	0 0 0	0 0 0	0 0 0				
Total										
Total in euros	2,331,596	3,962,152	45,859	5,506,485	182,355	275,280				
Mean per patient in euros (SD)	816.7 (997.8)	382.1 (536.4)	1,993.9 (4,008.2)	323.3 (435.0)	542.7 (702.5)	489.8 (837.3)	434.6	<.0001	493.3	<.0001
Median in euros Q1 Q3	596 413 855	285 182 436	294 184 1,070	249 153 390	385 235 599	352 241 536				

**Subgroup 1A** consisted of patients treated during the first postoperative month and who continued treatment continuously during the first postoperative year;

**Subgroup 1B** consisted of patients treated during the first postoperative month and who discontinued treatment during the first postoperative year;

**Subgroup 1Z** consisted of patients who were hospitalized with hypocalcemia <1.5mmol/l (ICD-10 code E8351) without any treatment during the first postoperative month;

**Subgroup 2C** consisted of patients untreated during the first postoperative year;

**Subgroup 2D** consisted of patients untreated during the first postoperative month, but who started treatment in the second or third postoperative month;

**Subgroup 2E** consisted of patients untreated during the first postoperative month, who started treatment after the third postoperative month

**1A vs 1B** : comparison between Group1A and Group 1B on mean costs per patient;

**1A vs 2C** : comparison between Group1A and Group 2C on mean costs per patient;

Δ : Average difference ; univariate analysis : comparison using non-parametric Wilcoxon test

Specific expenditures (after hospital discharge) were estimated for outpatients, except for rehospitalization for calcium disorder

Specific drugs included calcium, active vitamin D, magnesium, thiazide diuretics, phosphate binders; specific medical consultations or procedures included general practitioner, endocrinologist, general/visceral surgeon, ENT, plastic surgeon, rheumatologist, nephrologist, urologist, psychiatrist; specific bioassays included calcium, phosphorus, parathormone, 25-(OH)-vitamin D (D2+D3), plasma magnesium, urea, creatinine, urine calcium level, urine creatinine level, creatine clearance; specific imaging included urinary tract ultrasound, brain CT and/or MRI, skull X-ray and bone densitometry. Rehospitalization for calcium disorder included ICD-10 codes: E835, E8350, E8358.

**Table 6:** Incremental effects on overall costs (in euros).

	Model I (Group 1 vs Group 2)**		Model II (Subgroup 1A vs 2C)***	
	Incremental cost (in euros) (95% CI)	p-value*	Incremental cost (in euros) (95% CI)	p-value*
**Hypoparathyroidism**				
No hypoparathyroidism	ref		ref	
Hypoparathyroidism	142 (47-239)	0.003	776 (591-970)	<0.001
**Gender**				
Female	ref		ref	
Male	910 (784-1,040)	<0.001	916 (767-1,070)	<0.001
**Charlson comorbidity index**				
Charlson Index 0	ref		ref	
Charlson Index 1 or 2	1,299 (1,142-1,461)	<0.001	1,278 (1,090-1,474)	<0.001
Charlson Index > 2	5,239 (4,441-6,140)	<0.001	4,650 (3,778-5,658)	<0.001
**Ecological deprivation index**				
1st quintile (least deprived)	ref		ref	
2nd quintile	162 (17-312)	0.028	140 (-37-325)	0.124
3rd quintile	239 (87-397)	0.002	263 (74-460)	0.006
4th quintile	290 (135-451)	<0.001	363 (170-566)	<0.001
5th quintile (most deprived)	368 (206-535)	<0.001	310 (117-512)	0.002
**Type of thyroid resection**				
Total thyroidectomy	ref		ref	
Complex total thyroidectomy	1,630 (1,074-2,271)	<0.001	1,431 (787-2,201)	<0.001
Completion thyroidectomy	381 (230-538)	<0.001	437 (263-620)	<0.001
**Lymph node dissection**				
None	ref		ref	
Central compartment	1,163 (1,013-1,319)	<0.001	1,125 (939-1,320)	<0.001
Lateral compartment	1,863 (1,684-2,050)	<0.001	1,766 (1,546-1,997)	<0.001
**Cutaneous complications**				
No	ref		ref	
Yes	631 (73-1,298)	0.029	824 (122-1,703)	0.023
**Laryngeal complications**				
No	ref		ref	
Yes	1,045 (910-1,186)	<0.001	967 (802-1,138)	<0.001
**Hemorrhagic complications**				
No	ref		ref	
Yes	1,552 (1,130-2,021)	<0.001	1,600 (1,084-2,189)	<0.001
**Infectious complications**				
No	ref		ref	
Yes	2,541 (1,900-3,278)	<0.001	2,422 (1,667-3,319)	<0.001

**Group 1** consisted of patients with hypoparathyroidism;

**Group 2** consisted of patients without hypoparathyroidism or with indeterminate parathyroid status;

**Subgroup 1A** consisted of patients treated during the first postoperative month and who continued treatment continuously during the first postoperative year;

**Subgroup 2C** consisted of patients untreated during the first postoperative year;

* from a GLM model adjusting for age (<40yr, 40-51yr, 52-62yr, >62yr), gender, Charlson Comorbidity index, ecological deprivation index, types of thyroid resection, lymph node dissection and complications (cutaneous, laryngeal, hemorrhagic, infectious), year and region.

** Model 1 included Group 1 as patients with hypoparathyroidism and Group 2 as patients without hypoparathyroidism or with indeterminate parathyroid status

*** Model 2 included Subgroup 1A as patients treated during the first postoperative month and who continued treatment continuously during the first postoperative year and Subgroup 2C as patients untreated during the first postoperative year.

Similarly, the mean difference between patients in Subgroup 1A (permanent hypoparathyroidism) and patients in Subgroup 2C (no hypoparathyroidism) was €2,129.3 per patient (p<0.0001) for overall expenditures (**Table 5**), and €493.3 (p<0.0001) per patient for specific expenditures (**Table 6**).

After adjustment for potential confounders, the incremental cost for patients treated for a probable permanent postoperative hypoparathyroidism (Subgroup 1A) compared to patients without hypoparathyroidism (Subgroup 2C) was estimated at €776 (p<0.0001) (**Table 4**).

## Discussion - conclusion

The data presented here provide information on hypoparathyroidism patterns after total thyroidectomy for cancer during the first postoperative year, and assess the additional health care expenditures associated with the treatment of hypoparathyroidism on a nationwide scale in France.

Hypoparathyroidism was characterized by hospitalization for severe hypocalcemia at any time in the first postoperative year, a clear criterion of hypocalcemia and/or at least one delivery of calcium and/or vitamin D started within the first postoperative month without any further administration requirement, allowing us to capture different temporary patterns, in addition to permanent ones.

The rate of permanent hypoparathyroidism, measured at 9.1%, is of the order of most recent large series (2–4), such as the 2021 national audit run by the British Association of Endocrine and Thyroid Surgeons, which also reported a 9% rate in the cancer group (5). We also found, similarly to Villarroya-Marquina et al., that it was still possible for 23% (839/3694) of patients still treated for hypoparathyroidism at the sixth postoperative month to recover after the sixth postoperative month, which therefore does not seem to be the optimal cut-off to define permanent hypoparathyroidism (33). Finally, we found a higher rate of patients who had undergone cervical lymph node dissection (43%) in patients with hypoparathyroidism than in those who had no hypoparathyroidism (32%), which is consistent with other reports where lymph node dissection is considered as a risk factor for hypoparathyroidism (34).

In our study, after adjustment for potential confounders, the additional overall health expenditures for patients with hypoparathyroidism was €142 per patient from the payer’s perspective. This sum remains relatively modest, and possible explanations may be that our study did not take into account the cost of the initial surgery stay, and that, in the short-term, the difference in health care consumption between the groups pertains to inexpensive categories (drugs, laboratory tests, etc.). Health expenditures were estimated from the payer’s perspective (French national health insurance), and therefore did not include possible out-of-pocket expenses for the patient. These out-of-pocket expenses for patients were not estimated here. In any case, such an estimation would have been rough, as expenses not covered by the payer may be covered by complementary insurance not available in databases. Furthermore, drugs that are reimbursable but not presented for reimbursement or over-the-counter treatments represent out-of-pocket expenses but are not captured in the cohort. This unobserved part of consumption may vary according to the studied therapeutic class as mentioned by Bertocchio et al. (35).

We chose to assess the postoperative health care expenditure for all patients and for those suffering from hypocalcemia, regardless of duration, in order to gain an overall idea of what it represented on a national scale. As a result, we included patients with different hypoparathyroidism profiles, and therefore different expenditure profiles. The additional analysis of subgroups allowed a better description and understanding of these profiles.

It would seem, as a first approximation, that the majority of the expenditure in Group 1 originates from patients with permanent hypoparathyroidism (Subgroup 1A), but these results should be refined through a specific study of cost profiles based on the duration of hypoparathyroidism.

Finally, indirect costs (sick leave allowance and invalidity allowance) represented 55% of the additional cost in our study, which was unexpected. Although the impact of postoperative hypoparathyroidism on work has been qualitatively assessed (4, 15), the economic burden for National Health Care Insurance related to absenteeism has, to our knowledge, never been quantified. Future studies may help to shed additional light on this point. However, it is worth bearing in mind that this relatively large proportion relates to a somewhat modest additional cost, at least in the first year.

### Strengths of the study

Based on comprehensive real-world data, our study makes it possible to describe observed health expenditure without the need for extrapolation. Furthermore, by providing a comparison with a control group of untreated patients, i.e., without hypoparathyroidism, our study makes it possible to estimate postoperative hypoparathyroidism-related health expenditure. Finally, the nature of the data available makes it possible to categorize health expenditure, and to estimate not only specific costs directly associated with hypoparathyroidism, but also indirect health costs, thus making it possible to detect unforeseen expenditure. These three items offer new concepts compared to the literature. Wang et al. (10) and Nicholson et al. in Markov cohort model studies (12), and Mercante et al. in a randomized trial (13), compared the cost of several drug supplementation regimens for hypoparathyroidism, while Fanget et al., in a retrospective series, estimated an approximate additional management cost of hypocalcemia patients, including the initial surgical stay, and without specifying the perspective or the source of cost data (11). These studies varied in the items chosen to estimate the cost, whether it was the price of drugs (10–12), biological assays (10, 13), caregiver time (12, 13), excess days of initial hospitalization (11), or rehospitalizations (10), but none of these studies included a control group of non-hypoparathyroidism patients. Other studies did not include cost quantification, such as Chen et al., who reported, in a retrospective multicenter study, health resource utilization: pills and medical visits (14) and Siggelkow et al. who conducted a survey assessing, among other things, medication use, caregiver burden and impact on work in patients with hypoparathyroidism (15). These latter two studies did not specifically target postoperative hypoparathyroidism, and none of the studies mentioned above have specifically focused on cancer patients. Finally, Mathonnet et al., in a large study describing the complete care pathway of total thyroidectomy patients in France, also quantified the rate of hypoparathyroidism after total thyroidectomy for cancer, but did not provide any cost estimation (4).

### Limitations of the study

The inability to access patient records, which were anonymized, made it difficult to confirm the diagnosis of postoperative hypoparathyroidism. Rather than searching the SNDS database for patients with the ICD10 coding ‘hypoparathyroidism’, we preferred to use a more reliable definition of hypoparathyroidism based on automatically recorded drug reimbursement. With ICD10 codes, cases without hospitalization would not have been identified, only stays with hypoparathyroidism would have been located, but these codes do not provide sufficient clinical information, and they do not indicate the duration of the condition. Within the first postoperative month, these codes were found for only 21 patients: 19 in Group 1 and 2 in Group 2 (data not shown), therefore no bias was introduced. Even though laboratory tests are entered into the database (blood calcium, etc.), their results are not available, which implies the use of a proxy. We assumed that the initiation of vitamin D and/or calcium treatment immediately after total thyroidectomy, in previously untreated patients, left little doubt about the causal link between the treatment and the disease. However, we were interested to find that a group of patients (Subgroup 2D with n=336 patients), started their treatment after the first postoperative month. A further analysis of this small subgroup showed that one third of these patients (representing only 106 patients) started treatment after the first month, but continued it for the whole first year. These patients were most probably patients treated for permanent hypoparathyroidism, whose initial treatment was not been recorded, for example. Some patients started treatment after the third postoperative month, a timeframe that is inconsistent with primary hypoparathyroidism (Subgroup 2E with 562 patients, of whom 269 were still being treated at 1 year). Therefore, these were patients who were most likely treated for a reason other than postoperative hypoparathyroidism (renal osteodystrophy for instance), although it is theoretically possible that hypocalcemia requiring treatment could remain untreated for 3 months. However, given the low proportion of the potentially misclassified patient population, we consider that the use of the proxy we chose did not affect the findings of our study.

Finally, our study only considered the first postoperative year, because it corresponded to the first phase of the disease. This phase makes it possible to discriminate between temporary hypoparathyroidism and permanent hypoparathyroidism. After the first year, the occurrence of serious complications due to permanent hypoparathyroidism should allow the identification of the few cases potentially misclassified. These long-term complications can be expected to cause potentially significant additional costs. Therefore, a 5-year update of the current data has been planned.

In conclusion, our study found that 42.5% of patients who underwent total thyroidectomy for cancer in France were treated for postoperative hypoparathyroidism, and 9.1% remained treated one year post-surgery. During the first postoperative year, health expenditures were significantly higher for patients treated for postoperative hypoparathyroidism, even if the difference was small. A 5-year follow-up is planned to assess the more burdensome long-term complications and their costs.

## Data availability statement

The datasets presented in this article are not readily available because access to the data from the SNDS requires permission in accordance with Public Health Code (Articles L.1461-1 to L.1461-7) and French data protection act (loi n°78-17 janvier du 6 janvier 1978). Requests to access the datasets should be directed to lesdonnees@institutcancer.fr.

## Ethics statement

The studies involving human participants were reviewed and approved by French Data Protection Agency (Commission nationale de l’informatique et des libertés—Cnil (26)) n°2019-082 and 2019-083. Written informed consent for participation was not required for this study in accordance with the national legislation and the institutional requirements.

## Author contributions

FB, CB, SR, P-JB, and EB contributed to conception and design of the study. EB organized the database and performed the statistical analysis. FB wrote the first draft of the manuscript. EB and SR wrote sections of the manuscript. All authors contributed to manuscript revision, read, and approved the submitted version.
